# Molecular advances to study the function, evolution and spectral tuning of arthropod visual opsins

**DOI:** 10.1098/rstb.2021.0279

**Published:** 2022-10-24

**Authors:** Marjorie A. Liénard, Wendy A. Valencia-Montoya, Naomi E. Pierce

**Affiliations:** ^1^ Department of Biology, Lund University, 22362 Lund, Sweden; ^2^ Department of Organismic and Evolutionary Biology and Museum of Comparative Zoology, Harvard University, Cambridge, MA 02138, USA

**Keywords:** light-sensitive opsins, visual pigments, heterologous expression, comparative sequence analysis, protein modelling

## Abstract

Visual opsins of vertebrates and invertebrates diversified independently and converged to detect ultraviolet to long wavelengths (LW) of green or red light. In both groups, colour vision largely derives from opsin number, expression patterns and changes in amino acids interacting with the chromophore. Functional insights regarding invertebrate opsin evolution have lagged behind those for vertebrates because of the disparity in genomic resources and the lack of robust *in vitro* systems to characterize spectral sensitivities. Here, we review bioinformatic approaches to identify and model functional variation in opsins as well as recently developed assays to measure spectral phenotypes. In particular, we discuss how transgenic lines, cAMP-spectroscopy and sensitive heterologous expression platforms are starting to decouple genotype–phenotype relationships of LW opsins to complement the classical physiological-behavioural-phylogenetic toolbox of invertebrate visual sensory studies. We illustrate the use of one heterologous method by characterizing novel LW Gq opsins from 10 species, including diurnal and nocturnal Lepidoptera, a terrestrial dragonfly and an aquatic crustacean, expressing them in HEK293T cells, and showing that their maximum absorbance spectra (*λ*_max_) range from 518 to 611 nm. We discuss the advantages of molecular approaches for arthropods with complications such as restricted availability, lateral filters, specialized photochemistry and/or electrophysiological constraints.

This article is part of the theme issue ‘Understanding colour vision: molecular, physiological, neuronal and behavioural studies in arthropods’.

## 1. Opsins: spectral light-sensitive receptors

Animal visual opsins are a group of proteins that belong to the superclass of G-protein-coupled membrane receptors (GPCRs) and form functionally diverse light-sensitive photopigments [1,2]. Opsins are unique among GPCRs because each one permanently binds an inactive form of its specific ligand, rendering almost an instantaneous response upon photo activation [3,4]. Photosensitivity is enabled by the bound molecule or chromophore, *cis*-retinal (or a relative) typically derived from vitamin A [3]. The free retinal chromophore maximally absorbs photons of light in the ultraviolet (UV) around 380 nm but its sensitivity is shifted to react to higher energy, shorter wavelengths, when bound to a UV short-wavelength (SW) opsin, and to lower energy, longer wavelengths when anchored to a blue middle-wavelength (MW) or long-wavelength (LW) opsin [4,5].

The chromophore forms a covalent bond with a lysine residue (K) in the ligand-binding site of the opsin via a protonated Schiff base. This bond is conserved across all animal opsins and is stabilized by a nearby negatively charged residue, the counterion [4,5]. When the inactive *cis* form of the chromophore absorbs a photon of light, it changes to a new all-*trans*-retinal conformation. This, in turn, triggers a conformational change in the opsin coupled to a Gt protein in vertebrates or Gq protein in invertebrates, thus activating distinct downstream signalling cascades leading to the perception of light [3,6,7]. Since the chromophore-opsin complex (also called rhodopsin) is tuned to specific wavelengths of light, maximum opsin absorbance (*λ*_max_) represents a directly measurable phenotype [8,9]. Although recent studies have uncovered non-visual roles of traditional visual opsins [10], such as temperature discrimination [11] and gustatory functions [12], the strong connection between genotype and spectral sensitivity phenotype makes opsins a powerful model to understand phenotypic adaptation.

Opsins originated early in the evolution of metazoans and duplicated to give rise to three major gene groups: c-opsins and r-opsins, the main visual pigments of vertebrates and arthropods, respectively, and the less known retinal G-coupled receptors/Go opsins [1]. Whereas vertebrate visual pigments are Gt-coupled proteins that activate a transducin protein (Gt) cascade leading to a hyperpolarization response, invertebrate opsins are coupled to Gq proteins that initiate the phosphoinositol cascade, resulting in a depolarization response by the photoreceptor cells [1,7,13]. In addition to activating different phototransduction pathways, each group contains distinct opsin genes that give rise to visual pigments that evolved from a single ancestor via idiosyncratic gene duplications and absorb maximally at different wavelengths [1,7,13]. The ancestral repertoire of vertebrate visual c-opsins includes four gene families, the short-wavelength-sensitive SWS1 (UV, *λ*_max_ 344–445 nm) and SWS2 (blue, *λ*_max_ 400–470 nm), and the middle and long-wavelength-sensitive opsins, MWS (green, *λ*_max_ 480–530 nm) and LWS (red, *λ*_max_ 500–570 nm), respectively [14–16]. The arthropod visual r-opsins comprise five well-characterized families, including short-wavelength sensitive (SW), middle-wavelength sensitive (MW1 and MW2), and long-wavelength sensitive (LW1 and LW2) [6,17]. Ancestral insect lineages very likely harboured three opsin types, an SW (or UV), an MW (or blue) and an LW (or green) opsin, with gene expansions and/or losses accounting for the remarkable opsin diversity observed across extant insect groups [17,18]. In summary, vertebrate and invertebrate opsin families are functionally analogous, yet phylogenetically distantly related, having converged independently to absorb similar ranges of the light spectrum.

The primary factor determining the spectral sensitivity of a photoreceptor is the absorption spectrum of the expressed visual pigment [1,3]. Optical and electrophysiological factors such as screening and filtering pigments, as well as neural processing, can further shift the peak sensitivity of a photoreceptor to a particular wavelength of light [3,17]. In addition to lateral filtering tuning effects, the absorption maxima of visual pigments can be shifted by changing the chromophore type from the widespread retinal (A1) or hydroxyretinal (A3) to dehydroretinal (A2), which prompts the visual pigment to absorb longer wavelengths [3,13]. Another mechanism involves changing opsin amino acid residues found within or adjacent to the chromophore-binding pocket, shifting the chromophore's ability to absorb specific wavelengths of light [3,9,19]. This mechanism accounts for the vast diversity of spectral sensitivities useful for colour vision and results from the dynamic history of diversification and molecular evolution of opsins [1,3].

The striking phenotypic convergence in spectral sensitivities across major vertebrate and invertebrate opsin groups does not necessarily imply convergence of tuning mechanisms at the molecular level. Since amino acid substitutions of the key residues surrounding the chromophore have occurred during the evolutionary history of each lineage, distinct tuning mechanisms are likely to have modulated spectral sensitivity of different opsin groups [20,21]. Mechanisms underlying spectral tuning in vertebrate visual opsins have been characterized in detail, notably by means of spectroscopic analysis with recombinant proteins of wild-type and mutant opsins [22–25]. By contrast, the tuning mechanisms of invertebrate opsins are still largely unknown owing to the difficulty of obtaining sufficient yields of purified recombinant pigments for spectroscopic analyses, particularly for certain groups of opsins [20,26,27]. Nevertheless, recent years have seen remarkable progress in the availability of invertebrate genomic resources, in the improvement of bioinformatics and modelling tools, and principally, in the development of robust molecular assays to study spectral sensitivities of invertebrate visual pigments [20,27–30].

Here, we review these advances and summarize a framework for identifying and testing molecular variation relevant for spectral tuning in invertebrate opsins. We capitalize on some of these techniques and show their promise by characterizing sensitivities of LW opsins across diverse taxa, including a crustacean, an early divergent insect (a dragonfly), and several nocturnal lepidopteran species. Finally, we discuss the role of these methods in advancing knowledge on the mechanistic basis of functional convergence between vertebrate and invertebrate visual pigments and the evolutionary pathways modulating spectral tuning of colour vision genes.

## 2. Comparative sequence analysis of opsins

### (a) Mining, alignment and variation of opsin sequences

The lag in our understanding of tuning mechanisms of arthropod light-sensitive pigments compared to those of vertebrates is partly owing to the relative scarcity of genomic and transcriptomic data. However, the dramatic drop in sequencing costs has led to large-scale initiatives to sequence genomes and transcriptomes of thousands of arthropod species in the last decade or so [31–37]. Currently, more than 2700 assembled arthropod genomes are available via GenBank, with approximately 60% released in the last year. Pilot projects such as the Earth Biogenome project, the Tree of Life project by the Wellcome Sanger Institute, the i5 k initiative to sequence 5000 arthropod genomes, as well as the 1KITE to sequence more than 1000 insect transcriptomes have greatly improved data availability [31,32,36–38]. This abundance of new genomic and transcriptomic information has opened the door for studies of functional genomics of non-model organisms and comparative sequence analyses of opsins across a larger taxonomic breadth.

Once the genomic or transcriptomic resources have been found, three main approaches are routinely used to mine opsin sequences to identify orthologues and duplicated genes. These include sequence similarity, protein prediction through hidden Markov models (HMM), and phylogenetic inference [39–44] (table 1). The first two homology methods, sequence similarity and HMM profiles, can fall short in identifying opsins in distantly related species given the low similarity of some opsins (less than 50%) in different families and the high computational costs when analysing large datasets [49]. Conversely, phylogenetic approaches for functional annotation of opsins have been shown to overcome the shortcomings of homology-based methods [49,86]. In fact, the robust and efficient phylogenetically informed annotation (PIA) pipeline, primarily developed to mine for opsins, has recently been updated and used to explore patterns of opsin evolution across diverse arthropods groups such as amphipods, crustaceans, and butterflies and moths [49,86]. Alternatively, non-opsin light-sensitive GPCR proteins such as in the bay scallop, *Argopecten irradians,* have been recovered by combining RNA-sequence analysis and protein modelling workflows that target chromophore-binding lysine residues in positions that differ from the canonical lysine on the seventh transmembrane domain in opsins [87].

**Table 1.** Summary of steps for comparative analysis of opsin sequences, including selected programs and resources available for the different steps.

step	aim	suggested programs or pipelines
**mining and annotation**	retrieve opsin sequences	**programs:** BLAST [39], HMMER [40,45], GMAP [41], BLAT [42], AUGUSTUS [46]
		**pipelines:** BRAKER [47], MAKER [48], phylogenetically informed annotation (PIA) [8,49].
**identifying orthologues and paralogues**	discriminate duplicated genes	in principle, the resources used for mining and annotation can aid in finding duplicates, but some specific tools to discriminate orthologues from paralogs include OrthoFinder [50], i-ADHoRe [51], MCScanX [52], InParanoid [53].
**alignment and variant discovery**	characterize variation	**multiple sequence alignment:** MAFFT [54], Muscle [55], Clustal Omega [56].
		**mapping to reference sequence:** BWA [57], Bowtie [58], STAR [59].
		**variant calling:** GATK [60], bcftools [61], FreeBayes [62].
**ancestral sequence reconstruction**	reconstruct likely sequences at specific nodes	**tree inference:** IQ-Tree [63], RAXML-NG [64], PHYML [65].
		**ancestral reconstruction**: ANCESCON [66], PhyloBot [67], ProtASR2 [68], FireProt^ASR^ [69], FastML [70], PAML [71].
**selection analyses**	find residues under selection	***population level:* outlier analyses (OA):** Arlequin [72], FDIST [73], Bayescan [74].
		**environmental (EEA):** BAYENV/BAYENV2 [75], LFMM [76], RDA [77].
		***for diverged sequences:* dS/dN ratio:** PAML [71], HyPhy [78].
		**McDonald and Kreitman test:** iMKT [79]
**homology modelling**	identify candidate tuning sites through structure prediction	**programs**: Modeller [80], Swiss-Model [81], Phyre2 [82], AlphaFold [83],
		**visualization**: PyMOL [84], UCFS Chimera-A [85].

After retrieving candidate coding sequences, the second step is to spot genetic variation such as single nucleotide polymorphisms (SNPs), deletions, or insertions possibly linked to functional divergence. Although the direct association between phylogenetic groups and the capacity to absorb within a specific range of the spectrum make opsins amenable to functional classification solely based on similarity (UV, blue, LW), even moderate variation can translate into significant shifts in peak absorbance within groups [22]. To characterize variation among orthologues or duplicated genes, alignments are routinely performed using various algorithms (table 1). Beyond outlining variation between extant sequences, studies of vertebrate opsins have advocated for the use of ancestral sequence reconstruction (ASR) to infer the most plausible sequence at the root of the common ancestor between opsins of interest [88–90] (table 1). Identifying variants between ancestral and extant sequences has helped to reconstruct plausible scenarios for the historical accumulation of mutations and the order in which they appeared, as well as the evolutionary trajectories of spectral tuning of SWS1 visual pigments in vertebrates [23,24,91,92]. This approach, together with selection analyses, has been used to formulate hypotheses about opsin evolution in invertebrates, mostly Lepidoptera, Coleoptera and Hemiptera [93–96]. Nonetheless, candidate spectral tuning sites have seldom been validated, in sharp contrast to vertebrate opsins [27,29,30].

Most of the sequence variants identified in the previous step are likely to be synonymous changes or fall outside the binding pocket, thus playing a moderate part in generating opsin spectral diversity. The third step, therefore, is to narrow down the pool of variants to those that are more likely to be responsible for phenotypic changes in absorption maxima or protein stability, and thus might have been driven by or maintained by selection. Across species, rates of synonymous (dS) and non-synonymous substitutions (dN) and their ratio (dS/dN) are widely used to infer positive, negative or neutral selective pressures acting on opsin genes [97–99]. Using this method, studies have shown faster evolution of opsins in diurnal taxa, or more specifically, adaptive evolution in UV-sensitive opsins in day-flying insects and LWS opsins of day-flying Lepidoptera [6,96,100,101]. In addition, site-specific tests of dipteran, lepidopteran and stomatopod crustacean opsins have suggested positive selection at residues outside the chromophore-binding pocket; thus, hinting at adaptive roles potentially decoupled from spectral phenotypes [100,102,103]. At the population level, studies detecting genetic variants of opsins under selection have provided insights into the genetic basis of local adaptation to light environments and speciation [30,90,104,105]. The most widely used approaches to detect putatively adaptive SNPs at the population level are *F_ST_* outlier analysis (OA), which identifies variants with higher genetic differentiation among populations than expected under a neutral model, and environmental association analysis [106] (table 1). These approaches have been commonly used in vertebrates such as cichlids, guppies, sticklebacks, flycatchers and New World monkeys [107–111]. By contrast, only a handful of studies have characterized intraspecific variation of opsin genes in arthropods [30,112]. Consequently, little is known about the relationship between intraspecific variation and diversity of spectral sensitivities, and for the few examples known in arthropods, opsin diversification within species appears to be more consistent with natural selection unrelated to spectral tuning [30,112].

### (b) Protein modelling

Since visual opsins are also known to have adaptive extraocular functions [113], amino acid sites under selection may not be reliable predictors of variation in absorption maxima. Changes in the topographical distributions and orientation of the amino acid side chains surrounding the chromophore are primarily responsible for determining *λ*_max_ values, and thus building three-dimensional models of the visual pigments is essential [4,5,114]. In the absence of experimentally determined protein three-dimensional structures for the vast majority of opsins, homology modelling plays a cost-effective role [115]. The process begins by choosing the best template three-dimensional structure on which the target sequence can be successfully threaded [115]. For three-dimensional modelling of arthropod opsins, the squid rhodopsin template is preferred compared to the bovine rhodopsin because unlike the monostable and Gt-coupled visual opsins of vertebrates, squid and arthropod opsins are Gq-coupled and bistable, undergoing a 2-photon bidirectional photoreaction, with the retinal bound throughout the cycle [4,19,24]. The first crystal structure of a Gq-coupled opsin, jumping spider rhodopsin-1, was also published in 2019, providing additional insights into the molecular architecture of Gq-opsins and constituting a second available template for modelling arthropod visual pigments [4].

A series of alignments and alignment correction rounds are performed based on the template, followed by the generation of the backbone three-dimensional structure [115]. In opsin studies, the most frequently used tools have been the Swiss-Model and the Modeller program, which are knowledge-based evaluation methods, relying on scores representing energies obtained statistically within the context of all known experimental three-dimensional structures in the database [115] (table 1). For other photopigments with unknown homologous structures, machine learning-based methods such as AlphaFold2 [83] are expected to shed light on the molecular architecture of the visual pigments of non-model organisms or elusive families of opsins such as the non-visual groups. Finally, the predicted three-dimensional models and the chromophore are visualized to identify the amino acids lining in the binding pocket, which may be responsible for changes in *λ*_max_ values. As a rule of thumb, variants located less than or within 5 Å from the chromophore are spectral tuning sites candidates [116,117]. This is because 5 Å is the maximal length of weak hydrogen bonds, and the longest bonds between the visual pigment and the retinal that can alter the shape of the electrostatic environment within the binding pocket [116,117]. Although the variants surrounding the chromophore are expected to influence opsin tuning, ultimately, their role in modulating absorbance can only be tested experimentally.

### (c) Modelling approaches to predict peak spectral sensitivities

Site-directed mutagenesis of spectral tuning site candidates and *in vitro* experiments have convincingly linked amino acid changes to shifts in *λ*_max_ between opsin orthologues and duplicates, particularly in vertebrates [22,24,25]. Since the effects of some amino acid substitutions appear consistent across different vertebrate groups, these known spectral tuning sites have been used to infer *λ*_max_ based on sequence data [23,25,118]. For instance, the ‘five-sites’ rule posits that the identities of five critical sites within the binding pocket of some mammalian LWS can predict peak spectra [118]. Similarly, the A/B ratio, a ratio between the hydrogen-bond areas formed by five key amino acids of the SWS1 opsin in vertebrates, is shown to relate to variation in UV-violet perception [23]. More recently, through molecular dynamic simulations, Patel *et al*. [119] developed a statistical model able to predict *λ*_max_ values accurately for the blue opsin (Rh2) across teleost fishes. This study constituted an important leap forward in functionally predictive opsin modelling because it does not rely on data from laborious site-directed mutagenesis experiments, which have to account for additive as well as epistatic interactions. However, it is not clear whether this model extends beyond Rh2 opsins or to those from other animal taxa. In addition, although not requiring information about tuning residues, absorption maxima of the visual pigments are still necessary for model training. Thus, despite the potential for application of these methods to the prediction of *λ*_max_ of invertebrate opsins, comprehensive baseline information about experimentally characterized *λ*_max_ for purified invertebrate opsins remains largely limited. In the next section, we outline recent developments to characterize absorption spectra of Gq opsins *in vitro,* whose application to diverse organisms can fuel the development of predictive models tuned to arthropod visual pigments.

## 3. Molecular assays to elucidate absorbance maxima and tuning mechanisms

Molecular mechanisms that regulate invertebrate opsin absorption are still poorly understood [20,26]. However, in vertebrates, functional investigations of visual pigments have revealed critical mutations and amino acid substitutions responsible for adaptive shifts in absorption spectra [23,88,89,120]. This is predominantly because for the last 25 years or so, virtually any vertebrate opsin could be expressed and spectrally characterized *in vitro* [120]*.* In addition, vertebrate cells spontaneously bind 11-*cis*-retinal from solution, whereas for invertebrate opsins, it was not clear when this occurred or whether the retinal was eventually released [121]. Early attempts to evaluate the suitability of cell culture systems used in vertebrates to express invertebrate opsins, including mammalian COS1 cells, insect Sf9 cells, and amphibian *Xenopus* oocytes, failed to recover opsins capable of binding retinal [121]. At the time, these findings suggested that additional protein cofactors or chaperones for invertebrate photoreceptors might be necessary for the functional expression of visual pigments [121]. However, expressing opsin complementary DNAs (cDNAs) of other invertebrate species in transgenic *Drosophila* succeeded in producing visual pigments that bound retinal, showed normal spectral properties, and coupled to the endogenous phototransduction cascade [121–123]. Consequently, *in vivo* assays using *Drosophila* mutants became among the first methods available to study tuning mechanisms of arthropod opsins [21,30,121–123].

### (a) Transgenic opsin rescue lines using *Drosophila*

**Figure 1.**
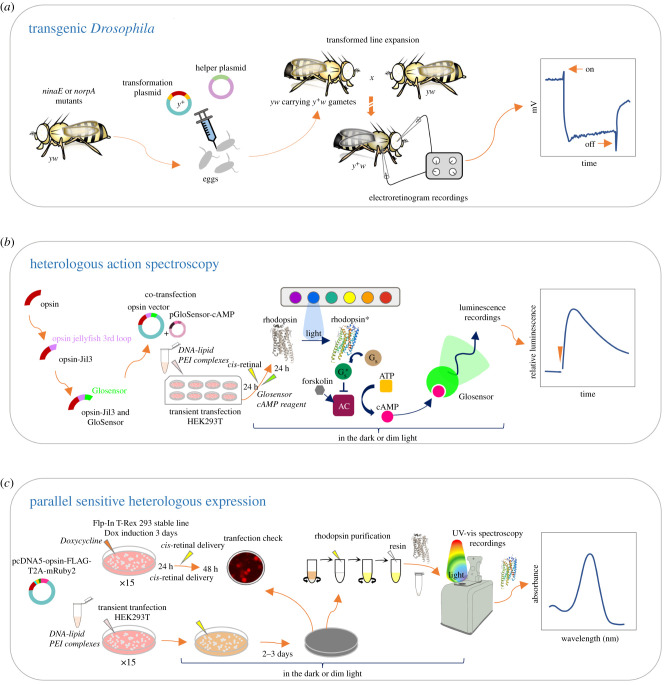
Overview of molecular methods used to analyse spectral sensitivities of invertebrate opsins. (*a*) *Transgenic Drosophila.* An expression plasmid carrying an opsin of interest under control of the Rh1, norpA or other promoter, is co-injected with a helper plasmid to control recombination and gene cassette insertion in the embryonic germ line pole. Injected developing flies are mated and their F_1_ progeny screened for the presence of positive transformants, typically via *y^+^w* phenotypes as schematized, or via mini-white *w^+^* eye marker phenotypes. The spectral sensitivity of fruit fly photoreceptors from final expression lines bearing opsin genes and markers of interest can be phenotyped by directly analysing pigment absorption of crude eye extracts [30] or via electrophysiology [21,122–125]. See text for details. (*b*) *Heterologous action spectroscopy (HeAS).* Gs-coupled opsins are engineered by replacing their third cytoplasmic loop with that of the Gs-couple jellyfish opsin. The opsin expression vectors are co-transfected with the pGloSensor cAMP plasmid. Following addition of 11, *cis*-retinal and pGloSensor reagents, and a 2-day opsin expression time course, light-induced changes in luminescence triggered by increased concentration of cAMP are measured by irradiation with lights of different spectral peaks. Modified based on [19,20,28]. See main text for details. (*c*) *Parallel sensitive heterologous expression (PaSHE).* Opsin open reading frames amplified from eye cDNA or synthesized for codon-optimization are individually ligated into a derivative of the pcDNA5 expression vector fused to a C-terminal epitope flanked by a peptide linker, a 21-amino acid long T2A cleavage site and a cytoplasmic fluorescent marker for visualizing cell transfection efficiency. Constructs can be transfected in the Flp-In Trex 293 cell line alongside a co-helper plasmid pOG44 to induce recombination and integration in the genome. Stable transformants are generated upon antibiotic marker selection (Hygromycin and Blasticidin), and opsin integration is validated through Sanger sequencing. A time course expression induction is then conducted in the presence of doxycycline, followed by quantitative polymerase chain reaction and western blot analyses (see the electronic supplementary material, methods). The stable validated line is expanded and Dox-induced in the presence of 11, *cis*-retinal prior to purifying the reconstituted visual pigments (see the electronic supplementary material, appendix). Stable lines can also be generated using alternative expression vectors, cell lines and/or epitopes (see [30,54]). Alternatively, the same expression vector can be used to transiently transfect HEK293T cells (24 μg DNA/plate at 4.10^6^ cells), prior to 11, *cis*-retinal delivery under dim light. Opsin expression and trafficking to the plasma membrane is typically detected within 2–3 days, which should be verified by fluorescent microscopy and western blot analyses prior to conducting large-scale purification. In both method variants, cells expressing opsins (induced or transient) are harvested, rhodopsin complexes are nutated with additional 11, *cis*-retinal and then solubilized from plasma membranes prior to purification by resin affinity, concentration and UV–vis spectroscopy recordings to obtain the visual pigment dark absorbance spectrum. Modified based on [29].

Early studies of opsin absorbance using *Drosophila* took advantage of the ‘blind’ *ninaE^17^* mutant flies, which lack Rh1, the gene encoding the visual pigment expressed in the major class of photoreceptor cells [122]. Accordingly, the expression of functional opsins in these mutants rescued light response fully [122]. Thus, the opsin of interest, or modified versions by site-directed mutagenesis could be subcloned into an expression cassette containing the native *Drosophila* Rh1 promoter [21,122,123]. This construct is introduced into the genome of *ninaE^17^* by *P*-element mediated germline transformation, and spectral sensitivities are then measured by electrophysiological analyses with electroretinogram recordings of live flies exposed to different wavelengths of light [21,122,123] (figure 1*a*).

Unsurprisingly, the spectral properties of different *Drosophila* visual pigments were among the first to be characterized in arthropods, followed by opsins of the honeybee and *Limulus* horseshoe crabs [121,122,124]. The first study that mutated insect visual pigments was conducted using this method*.* Salcedo *et al*. [123] studied spectral tuning of *Drosophila* blue and UV receptors (Rh1 and Rh3, respectively) (2003) [123], and later the LWS (Rh6) (2009) receptor [21]. They identified the variants UV: lysine versus blue: asparagine or glutamate (G90 in bovine rhodopsin system) that shift the absorption of the blue (Rh1) pigment into the UV, as well as the serine/alanine substitution (Ala-292 in bovine rhodopsin) residues implicated in changing the absorption of LWS (Rh6) to shorter wavelengths [21,123]. Interestingly, this site (Ala-292, in bovine rhodopsin) is also responsible for regulating LWS sensitivity in birds and mammals, thus providing the first example of a convergent tuning mechanism between vertebrate and invertebrate visual pigments [21]. More recently, the *in vivo* spectral sensitivity of *Drosophila* photoreceptors was re-examined by selectively restoring photoreceptor activity in flies with otherwise no receptor activity (*norpA* mutants) [125]. Sharkey and colleagues engineered single-opsin rescue fruit fly lines expressing phospholipase C (PLC, encoded by *norpA*) under the control of a specific opsin promoter, which restored sensitivity of each pigment in its native photoreceptor cell surrounded by its native lateral pigment [125]. This approach makes it possible to express and record electroretinogram (ERG) activity from mutant opsins in a natural photoreceptor environment.

### (b) Towards the development of heterologous systems

Despite the significant contributions of transgenic *Drosophila* methods to the early understanding of spectral tuning of invertebrate opsins, these *in vivo* assays made it difficult to disentangle the contributions of filtering and screening pigments to the spectral properties of photoreceptors from that of pure opsins, although the use of white-eye (*w*) mutants tends to reduce these effects. To understand the molecular evolution of spectral tuning sites, direct analysis of purified visual proteins is indispensable [20,26]. However, studies exploring the molecular bases of spectral tuning in invertebrate opsins remained limited by the lack of an optimized *in vitro* expression system that would yield sufficient active rhodopsin complexes comparable to those recovered for vertebrate opsins [20,26].

#### (i) Short-wavelength opsin expression systems

Terakita and colleagues in 2008 were the first to describe heterologous expression of a Gq-protein-coupled opsin in cultured cells [26]. Unlike previous attempts, they used the human embryonic kidney (HEK) 293 cell system, which was a cultured tissue not surveyed before by the Knox *et al*. study [121]. Terakita *et al*. [26] successfully obtained protein yields of UV, blue and LWS opsins of the Japanese honeybee, the first arthropod opsins ever expressed in cultured cells [26]. However, although the expression of the three proteins was detected, pigment formation was not observed for the LWS opsin, suggesting that folding of this protein did not occur [26]. Similarly, they were unable to obtain active proteins for other Gq-coupled opsins such as those for molluscs and crustaceans, leading them to suggest that higher thermal stability or the need for other co-expressed chaperones might be required to obtain functional LWS opsins and other unsuccessfully expressed pigments [26].

Building upon the Terakita *et al*. [26] HEK cell assay, which proved to be efficient for studying opsins absorbing in shorter wavelengths [26], Wakawuka and colleagues [27] subsequently studied blue duplicated opsins of the cabbage white butterfly *Pieris rapae* [27]*.* They reconstituted the blue and violet receptors *in vitro* using HEK293 cultured cells and used spectroscopy to characterize absorption maxima [27]*.* Through site-directed mutagenesis, they found two amino acid substitutions between the duplicate copies crucial for shifting spectral sensitivity and whose positions suggested a tuning mechanism specific to invertebrates [27]. Frentiu *et al*. [30] also used HEK293 cultured cells to study SW opsins but focused on the role of intraspecific variation in contrast to variation between duplicated opsins [30]. They developed a first stable cell culture system for the expression of insect opsins to test the effect of clinal variation on spectral tuning of the blue opsin of *Limenitis arthemis* populations across a latitudinal gradient [30]. Stable cell lines can carry a genetic modification resulting in constant and modulable expression levels, which, if the opsin folds properly, yield higher protein levels for accurate measurement of *λ_max_* [30]. In summary, until recently *in vitro* studies of arthropod opsins primarily allowed reconstituting visual pigments for SW and MW opsins from the UV and blue opsin families.

#### (ii) Long-wavelength opsin expression systems

LW pigments were believed to be difficult to express in cultured cells on a large scale until optimized assays emerged to circumvent this problem [20,29]. To the best of our knowledge, two methods have been successful at consistently characterizing absorption spectra of arthropod LW opsins *in vitro*: heterologous action spectroscopy (HeAS), and parallel sensitive heterologous expression (PaSHE) [20,29].

*Heterologous action spectroscopy.* This first method was initially implemented to study non-visual opsins, such as Opn3, with unknown signalling cascades [19,28]. The first arthropod opsin whose spectral sensitivity curve was accurately estimated using heterologous action spectroscopy was the Gq-coupled jumping spider Rh1 (UV) [19,28]. More recently, Saito *et al*. [20] used HeAS to pinpoint the main helices and residues therein responsible for spectral tuning of duplicated LW opsins of the butterfly *Papilio xuthus,* thus showing that this method is not only functional for UV visual pigments but also for studying LW opsins [20].

Heterologous action spectroscopy is based on the quantification of light-dependent changes in the cyclic second messenger of cultured cells expressing opsins of interest [20,28] (figure 1*b*). Since arthropod Gq-coupled visual opsins drive phosphatidylinositol-related cascades instead of cyclic nucleotides cascades like vertebrate Gt-coupled, Gs, Gi and Go-coupled cnidarian opsins, which use the cyclic nucleotides cGMP and cAMP as second messengers in the phototransduction cascade [7,19], the first step to enable heterologous action spectroscopy is to engineer Gs-coupled versions of the Gq-coupled opsins by replacing the third cytoplasmic loop with that of the Gs-coupled jellyfish opsin by polymerase chain reaction [20,28]. The third intracellular loop is the one replaced because it is known to be a major determinant of G protein selectivity in opsins [19]. By changing its third loop, an opsin can be expressed *in vitro* even if the G-protein with which it couples is unknown [28]. The chimeric Gs-version of the protein of interest is transfected into HEK293 cells with the GloSensor plasmid, which codes for a cAMP-sensitive luciferase [20,28] (figure 1*b*). Upon activation with light stimuli, changes in cAMP levels are measured based on the luminescence derived from the GloSensor luciferase protein [20,28] (figure 1*b*). Light sources with different spectral emission peaks (from 410 nm up to 630 nm) are used for the estimation of wavelength-dependent changes in intracellular cAMP in response to each light stimulus [20,28] (figure 1*b*). To calculate relative sensitivities, the amplitude of these wavelength-dependent responses are extrapolated using a light intensity dose–response curve generated by quantifying responses to a single light stimulus (green 500 nm, orange 600 nm) while varying the intensity [20,28]. Finally, the absorption spectrum is estimated by fitting these relative sensitivities to a rhodopsin template [20,28].

**Figure 2.**
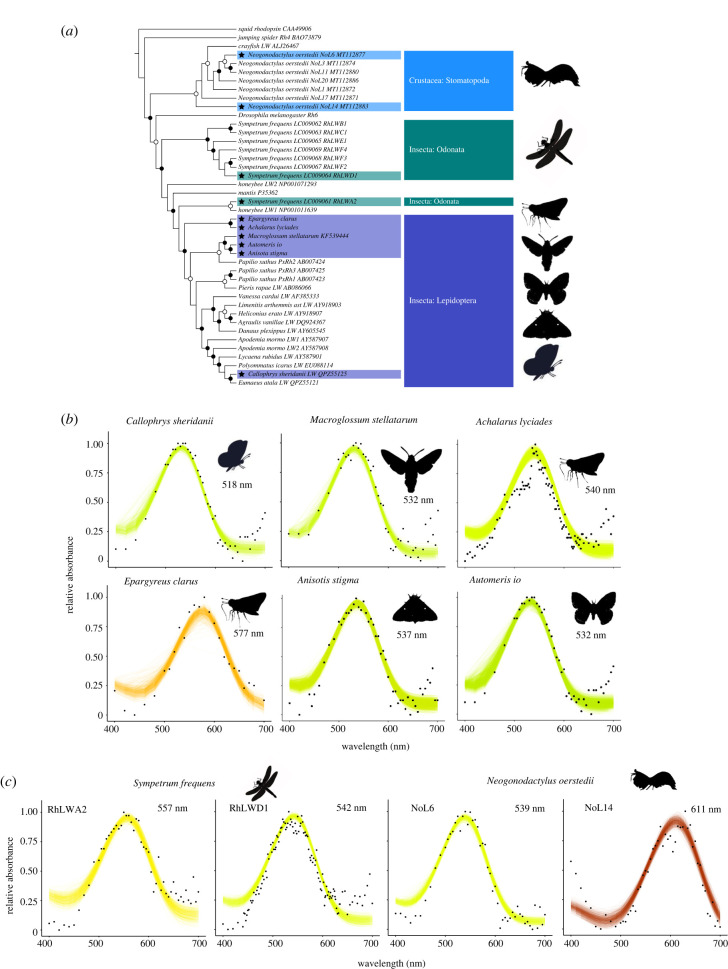
Functional expression of insect and crustacean long-wavelength (LW) opsins. (*a*) Maximum-likelihood phylogeny of LW opsins in exemplar taxa from Insecta, Arachnida and Crustacea. Branch names comprise the full species name, the GenBank accession number and sequence acronym. LW opsins functionally characterized in this study are labelled with a black star. (*b,c*) Dark absorbance spectra of long-wavelength rhodopsin visual pigments reconstituted and purified from cell cultures in the presence of 11-*cis*-retinal using the PaSHE workflow. (*b*) Dark absorbance spectra of selected long-wavelength lepidopteran insect opsins. (*c*) Dark absorbance spectra of selected long-wavelength odonate insects and stomatopod crustacean opsins. The black dots represent mean absorbances. Absorbance at 380 nm when present, is owing to residual unbound *cis*-retinal. Relative absorbance data are fitted to a visual template [127] with polynomial function analyses computed in R to obtain the best estimates of lambda max for each opsin. Number of protein eluate aliquot measurements (*n*): *Callophrys sheridanii* (*n* = 4), *Macroglossum stellatarum* (*n* = 6), *Automeris io* (*n* = 2), *Anisota stigma* (*n* = 3), *Achalarus lyciades* (*n* = 4), *Epargyreus clarus* (*n* = 2), *Sympetrum frequens* (*n*_RhLWA2_ = 3, *n*_RhLWD1_ = 9), *Neogonodactylus oerstedii* (*n*_NoL6_ = 9, *n*_NoL14_ = 14).

*Parallel sensitive heterologous expression*. PaSHE is an *in vitro* assay that allows efficient purification of arthropod visual opsins for spectroscopic analysis [29]. PaSHE is essentially a heterologous expression system that builds upon previous protocols used to express opsins in HEK293T [26,27,30,87], but through a series of optimized steps is able to consistently recover active rhodopsin complexes for the different Gq opsin families, including the elusive LWS [29]. Synthetically codon-optimized opsin sequences are effectively expressed, although constructs using native sequences work comparably well depending on the species investigated. Briefly, this simple system uses an expression cassette under a strong cytomegalovirus promoter, engineered to contain a mammalian transcription sequence upstream of the opsin start codon, and fused in 3′ with a Flag epitope [29]. The Flag epitope is directly flanked by a T2A peptide and a cytoplasmic fluorescent mRuby2 marker (figure 1*c*). The T2A peptide contains a Proline residue that mediates cleaving of the co-translated proteins [126]. Small-scale transfections and western blot are performed to select high expression plasmids, followed by large-scale purification. Transfected plates are supplemented with *cis-*retinal after 6 h and incubated in the dark for at least two nights to maximize membrane protein expression. Cells are harvested, and opsin-FLAG proteins are solubilized from plasma membranes then separated from the crude extract using FLAG resin and a subsequent column purification prior to competitive binding with FLAG-peptide. Finally, rhodopsin complexes are eluted and concentrated by centrifugation. Dark absorbance spectra of concentrated opsins are measured using UV–Vis spectroscopy, followed by estimation of *λ*_max_ by fitting the raw data to a visual template (figures 1*c* and 2*b,c*).

The methods described above to characterize *λ*_max_ *in vitro* open the door to dissect LW opsin tuning mechanisms and to start answering long-standing questions about the genetic basis of functional convergence between invertebrate and vertebrate LW opsins. For example, in a series of elegant chimeric and site-directed mutant experiments, Saito *et al.* [20] used HeAS to successfully narrow down two single mutations in helix III, A116G (+6 nm) and F120Y (+3 nm), that synergistically contribute to a +15 nm shift between duplicated PxRh1 (540 nm) and PxRh3 (570 nm) *Papilio* opsins [20]. By contrast, two sites in helices IV and VI in LW duplicated opsins in primates are the main contributors to their 30 nm difference in *λ*_max_; thus suggesting different mechanisms underlying similar shifts in absorbance in primates and insects [20]. However, it is not clear how general the LW tuning mechanism observed in *Papilio xuthus* is across butterflies and more generally, across arthropods. A first step towards investigating the potential diversity of spectral sensitivities and tuning mechanisms of LW across arthropods is to increase the sampling and characterization of LW opsins, especially for independently duplicated opsins. In the next section, we present new data for LW absorption spectra across Lepidoptera and other arthropod groups to show the range of spectral sensitivities spanned by this gene family and as a baseline for studying independently evolved LW duplications in invertebrates.

## 4. Functional insights into arthropod long-wavelength opsin sensitivities

To expand the range of PaSHE applications to study Gq opsins of more distantly related arthropod groups and gain insights into spectral sensitivities of invertebrate LW opsins, we analysed 10 additional LW opsins across two insect orders and a crustacean. In this study, we aimed to encompass phylogenetic and ecological diversity by comparing: (i) diurnal and nocturnal lepidopteran species, (ii) species with terrestrial and/or aquatic lifestyles, and (iii) species with a large number of duplicated LW opsins (figure 2*a*). For each LW opsin, we used PaSHE to obtain dark absorbance spectra from purified LW Gq opsins (figure 2*b,c*; electronic supplementary material, dataset S1, SI appendix, figures S1 and S2), including six lepidopteran species from four families (Lycaenidae, Hesperidae, Sphingidae and Saturniidae) (figure 2*b*), a dragonfly, *Sympetrum frequens* (Odonata) and a stomatopod crustacean (*Neogonodactylus oerstedii*) (figure 2*c*).

Insect LW photoreceptors are known to harbour a large amount of spectral variation, accounting for rhabdomeric filtering pigments, functional variation in the expressed visual pigments and visual pigment co-expression, altogether leading to LW spectral sensitivity peaks ranging from 500 to above 600 nm [17,128]. We show that variation at the photoreceptor level is mirrored by maximal peak sensitivities of individual rhodopsins (figure 2*b,c*; electronic supplementary material, dataset S1). The reconstituted LW opsin from *Callophrys sheridanii* absorbed maximally at 517.8 nm. The LW opsin from the diurnal hawkmoth *Macroglossum stellatarum* absorbed at 531.7 nm, a peak absorbance a few nanometres above previous ERG recording estimates (521 ± 3.6 nm [127]), possibly owing to the optics of hawkmoth eyes and/or to using A1 chromophore instead of the native A3 retinal. LW opsins from diurnal and nocturnal saturniid moths, *Anisota stigma* and *Automeris io*, absorbed maximally at 537 nm and 532 nm, respectively. The two opsins share a high level of sequence similarity with only 11 variant residues, which our homology modelling predicts to fall outside candidate sites interacting with the chromophore. The close absorbance peaks are thus expected and support the reliability and reproducibility of the method in obtaining precise estimates of *λ*_max._

Skippers (Hesperiidae) are unusual among butterflies for possessing eyes similar to the superposition eyes more typical of diurnal moths. While it remains a challenge to conduct *in vivo* studies of spectral sensitivities of visual pigments for superposition eyes, *in vitro* assays have the advantage of being effective regardless of eye morphology. We cloned an LW opsin *de novo* from the eye cDNA of a grass skipper *Epargyreus clarus* (Eudaminae), which has a single LW opsin gene, similarly to *Lerema accius* (Hesperiinae). Taking advantage of the high-quality genome of the Hoary edge, *Achalarus lyciades* (Hesperidae, Eudaminae) [129], which inhabits open woodlands, forest edges and roadsides, we additionally mined its duplicated LW1 and LW2 opsin gene sequences (see methods). Of these, we chose to purify the paralogues *A. lyciades* LW2 and *E. clarus* LW opsins, which we found absorb maximally at 540 and 577 nm, respectively. These results illustrate the potential to explore visual gene functions from a genomic and functional angle, and to infer patterns regarding how ecological specializations might be correlated with adaptations in peripheral light-sensing genes.

For the LW opsin in the skipper butterfly *E. clarus* (figure 2*b*), we generated a Doxycycline-inducible stable line using the Flp-In TRex system (Invitrogen) as described in the electronic supplementary material, methods. The recombinant opsin expressed well from 3 days post-induction (electronic supplementary material, figure S2), a time point selected for purification. From spectroscopy measurements, we observed that the recombinant rhodopsin complexes have a two to threefold higher absolute absorbance (electronic supplementary material, dataset S1) consistent with earlier absorption measurement levels obtained with a non-inducible stable cell line expressing *Limenitis a. astyanax* BRh [30]. Establishing stable cell lines, however, takes time, requiring on average 30 days before obtaining stable polyclonal lines, plus additional time to validate the line and test optimal gene and protein expression time course. Alternatively, using a high-expression cytomegalovirus promoter together with native full-length transiently expressed opsins represents a time efficient, scalable method for parallel purifications, which is adaptable to all arthropod Gq opsins. Cloning opsin sequences sequentially de novo or reamplifying open reading frames from fresh biological material may also be replaced by synthetic codon-optimized sequences, providing a time and cost-effective option further maximizing high protein expression levels.

**Figure 3.**
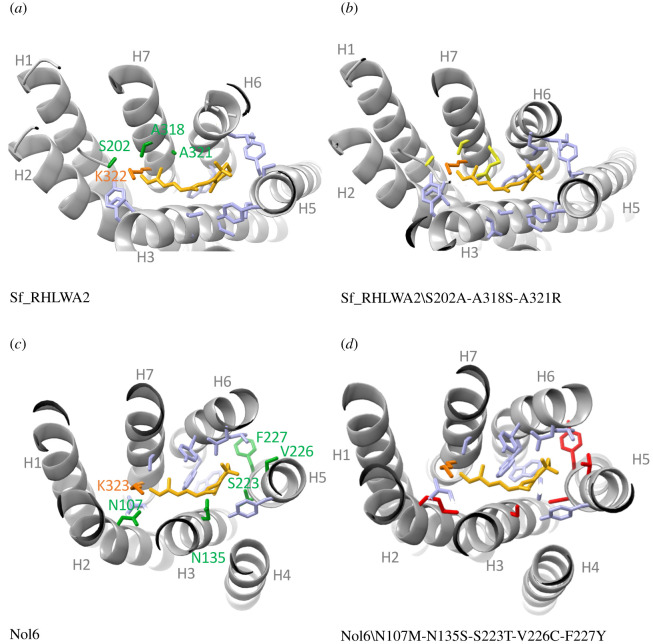
Example of homology modelling to identify tuning sites among duplicate LW Gq opsins. (*a*) Structure of the dragonfly *Sympetrum frequens* Sf_RhLWA2 opsin built against homology modelling with the jumping spider crystal structure (PDB 6i9k). Among 127 variant sites with Sf_RhLWD1, three variant sites are within 5 Å of the *cis*-retinal chromophore. Helices are represented in light grey, the *cis*-retinal is represented in yellow, residues predicted to interact with the chromophore are in light blue (electronic supplementary material, dataset S3). Among these, the three variant candidate spectral sites are coloured in green. (*b*) Sf_RhLWA2 structure with the three variant sites mutated to those of Sf_RhLWD1 and coloured in yellow. (*c*) Structure of the green-sensitive NoL6 LW Gq opsin of the stomatopod *Neogonodactylus oerstedii* modelled against 6i9k. Among 155 variant sites with the red-shifted sensitive NoL14 opsin, five candidate spectral sites, coloured in green, are found in the vicinity of the chromophore. (*d*) NoL6 structure with mutated residues corresponding to NoL14 at each candidate spectral site. The templates and models were built in SwissModel followed by visualization in PyMol.

Most methodological advances to study arthropod LW opsins *in vitro* have focused on butterfly species. Thus, an outstanding question is whether paralogous LW opsin genes provide similar potential variability in their corresponding peak spectral sensitivities across more distantly related lineages*.* To explore this question, we experimentally measured peak spectral sensitivities of duplicated opsins in the autumn darter dragonfly, *Sympetrum frequens* (Sf) and the stomatopod crustacean, *Neogonodactylus oerstedii* (No), reconstituted with *cis*-retinal (figure 2*c*), a required step prior to inferring the ancestral sequences for extant LW opsins in these arthropod lineages, as well as their evolutionary trajectories and timing of spectral substitutions. We observed that Sf_RhLWD1 and Sf_RhLWA2, opsins expressed in the dorsal and ventral adult eyes across at least 12 surveyed dragonfly species [130] have peak absorbances for green wavelengths at 542 nm and 557 nm, respectively (figure 2*c*). Homology when compared with the jumping spider rhodopsin structure provided a reliable model, recovering all key conserved sites known to interact with the chromophore (lysine, counterion and glycines). Among 127 variant amino acid positions, three amino acids are within 5 Å of the vicinity of the chromophore (electronic supplementary material, dataset S3), including an Sf_RhLWD1-Alanine to Sf_RhLWA2-Serine substitution in EL2, which is known to cause shifts (towards longer wavelengths), and represents a robust candidate for *in vitro* site-mutagenesis experiments (figure 3*a,b*).

For the stomatopod, *N. oerstedii*, phylogenetic and *in situ* hybridization expression patterns previously showed that NoL6 and NoL14 belong to distinct phylogenetic opsin subgroups (A and F) known to be expressed in separate LWS photoreceptive units [102,131]. NoL6 is expressed in the mid-band eye region in ommatidial rows 5 and 6, also known to be tuned for polarization vision [132] and NoL14 is expressed across all seven (R1-R7) photoreceptive units in the dorsal and ventral eye hemispheres, but not in the mid band region [102]. Our proof-of-concept results show that both constructs formed active rhodopsin complexes in cultured cells in the presence of *cis*-retinal, with each purified visual pigment complex exhibiting maximal absorbance peaking at 539 nm (NoL6) and 611 nm (NoL14), respectively (figure 2*c*). The recorded peak sensitivities of stomatopod crustacean photoreceptors are notoriously variable resulting from rhabdoms divided into tiered layers, each expressing one or several distinct opsin visual pigments, alone or in addition to photostable filter pigments [132], which together produce up to 16 spectral receptor classes across the eye [102]. Opsin *in vitro* sensitivities are in line with the finding that NoL6 is co-expressed with a candidate middle-wave-sensitive (MWS) opsin, NoM10, resulting in photoreceptor sensitivity near 520 nm in mid-band rows 5 and 6 [102,132]. Although it is challenging to compare *in vitro* to microspectrophotometry estimates because of co-expression, our findings provide preliminary support for the functional distinctiveness of co-expressed LWS opsins. Although the two opsin proteins characterized here were selected in part because they are known to differ in expression patterns, phylogenetic distances and sequence (NoL6 and NoL14 share 69% identical residues and contain 155 amino acid variants), we identified a minimal number (five) of candidate spectral substitutions (electronic supplementary material, dataset S3) out of 30 previously predicted sites [102] that could underlie transitions from green to red-shifted absorbing opsins and can be readily tested *in vitro*.

## 5. Conclusion

In this essay, we compare and contrast available methods to gain insights into the diversity and function of invertebrate Gq rhodopsins. A combination of reproducible bioinformatic, modelling and heterologous methods are now available to characterize sequence variation and spectral sensitivity of virtually any invertebrate Gq opsin. These represent advances on multiple fronts: first, the growing availability of opsin sequences and bioinformatic tools allow for identification of residues under selection and systematic characterization prior to experimental testing of candidate tuning sites and/or duplication events. Second, *in vitro* methods such as HeAS and PaSHE make it possible to isolate the contribution of single pigments to visual sensitivity across invertebrate groups with complex visual systems and photoreceptor classes. As opsins show a strong connection between genotype and phenotype, these workflows pave the way to larger scale evolutionary and comparative studies to reveal the mechanistic basis of visual adaptation across arthropods. These methods now allow us to retrace spectral tuning trajectories to elucidate the genetic basis of functional convergence and the role of epistasis, mutation and mutation-order accumulations in shaping spectral phenotypes.

This review focuses on methods to investigate visual opsins, as they are the most comprehensively studied to date. Nonetheless, these methods have the potential to be easily extended to examine the functions of extraocular opsins. In fact, HeAS was primarily developed to evaluate the light-dependency of vertebrate non-visual opsins expressed in the brain [28]. Similarly, heterologous systems expressing the non-visual Rh7 opsin were used to characterize their role as circadian photoreceptors in the brain [133,134]. Other non-canonical functions of opsins in thermosensation, hearing and taste are more challenging to research, although, in principle, these *in vitro* systems could be adapted with other recording assays to test a range of ligands and responses.

A remaining challenge to these methods is the speed and efficiency of screening for the presence and function of different variants. The number of experiments grows exponentially with the number of candidate spectral sites and their potential interactions, placing constraints on the number of opsin variants that can be characterized simultaneously. Nevertheless, insights gained from being able to characterize LW opsins with spectral sensitivities absorbing far in the red have already brought promise for a diversity of new applications in optogenetics, which in turn can fuel the development of high throughput methods to screen arthropod visual opsins. Robust workflows to survey the diversity of opsins across arthropods provide the critical first step to uncovering the repertoire of GPCR opsins and thereby expanding our understanding of the full palette of wavelengths that are perceived and used by organisms across the Tree of Life.

## 6. Methods

### (a) Phylogenetic analyses

Predicted LW opsin amino acid sequences from selected arthropod species were retrieved from GenBank, aligned using MAFFT [54] computed with IQ-Tree [135] with LG + F + G4 as the best-fit substitution model [136], using the SH-aLRT branch test [137] and visualized using EvolView [138]. The *Achalarus lyciades* LW opsin sequence was retrieved from genomic data [129] (MOOZ01000541, scaffold887_len869015_cov66) and annotated using AUGUSTUS [46]. LW opsin sequences from *Callophrys sheridanii*, *Anisota stigma*, *Automeris io* and *Epargyreus clarus* were characterized from eye messenger RNA following a protocol previously described for *Arhopala japonica* [29].

### (b) Heterologous expression and purification of insect and crustacean opsin genes

To express the *E. clarus* LW opsin, we generated a doxycycline-inducible stable line using the native cDNA sequence as detailed in the electronic supplementary material, methods, figure S1, dataset S2). For other LW opsins, native open reading frames were amplified from cDNA (for *C. sheridanii*, *A. io* and *A. stigma*), or retrieved from GenBank with accession numbers listed in the electronic supplementary material, dataset S1 and codon-optimized sequences were synthesized by Genscript, with restriction sites suitable for subcloning in pCDNA5-FLAG-T2A-mRuby2, then overexpressed by transient transfection in HEK293T following the PaSHE procedure [29] followed by purification, spectroscopy and western blotting (electronic supplementary material, methods, figure S2). For each rhodopsin, we obtained estimates of *λ*_max_ through nonlinear least-square fitting to the absorbance data according to the template formula proposed by Govardovskii *et al*. [139]. We performed 1000 bootstrap replications to calculate *λ*_max_ predictions and confidence intervals in R v. 3.6.6 using the packages *rsample* and *tidymodels*.

## Data Availability

The opsin sequence data for *A. stigma*, *A. io* and *E. clarus* are available in GenBank under accession nos OK930068–OK930070. Source data and code underlying functional expression, qPCR analyses and homology modelling are available as electronic supplementary material files [140].
